# Ibrutinib increases the risk of hypertension and atrial fibrillation: Systematic review and meta-analysis

**DOI:** 10.1371/journal.pone.0211228

**Published:** 2019-02-20

**Authors:** Daniel Caldeira, Daniela Alves, João Costa, Joaquim J. Ferreira, Fausto J. Pinto

**Affiliations:** 1 Serviço de Cardiologia, Hospital Universitário de Santa Maria (CHLN), CAML, Centro Cardiovascular da Universidade de Lisboa—CCUL, Faculdade de Medicina, Universidade de Lisboa, Avenida Professor Egas Moniz, Lisboa, Portugal; 2 Laboratório de Farmacologia Clínica e Terapêutica, Faculdade de Medicina, Universidade de Lisboa, Avenida Professor Egas Moniz, Lisboa, Portugal; 3 Instituto de Medicina Molecular, Faculdade de Medicina, Universidade de Lisboa, Avenida Professor Egas Moniz, Lisboa, Portugal; 4 Hematology Department, Faculdade de Medicina, Universidade de Lisboa, Santa Maria University Hospital (CHLN), Lisbon, Portugal; Institute for Quality and Efficienty in Health Care (IQWiG), GERMANY

## Abstract

**Introduction:**

Ibrutinib is an oral covalent inhibitor of Bruton's tyrosine kinase approved for the treatment of patients with chronic lymphocytic leukemia (CLL), mantle cell lymphoma and Waldenstrӧm’s macroglobulinemia. Ibrutinib has an increased risk of atrial fibrillation but the mechanism is unknown, and hypertension may play a role in the pathogenesis of this adverse drug reaction.

**Methods:**

We aimed to review the risk of hypertension and atrial fibrillation as adverse events associated with ibrutinib through a systematic review with meta-analysis of randomized controlled trials (RCTs) retrieved in December 2018 on MEDLINE, EMBASE, CENTRAL and ClinicalTrials.gov. The data were pooled using random-effects meta-analyses using the risk ratio (RR) with the 95% confidence interval (95%CI). The confidence on the pooled estimates was ascertained through the grading of recommendations assessment, development, and evaluation (GRADE) approach.

**Results:**

There were 8 eligible RCTs (2580 patients), all reporting safety data of interest. Ibrutinib was associated with a significant increase in the risk of hypertension with a RR of 2.82 (95%CI 1.52–5.23) with moderate quality evidence. Ibrutinib increased significantly the risk of atrial fibrillation with a RR of 4.69 (95%CI 2.17–7.64) with high quality evidence.

**Conclusions:**

Ibrutinib was associated with significantly increased risks of both hypertension and atrial fibrillation.

## Introduction

Ibrutinib is the first-in-class oral covalent inhibitor of Bruton's tyrosine kinase that has been approved for the treatment of patients with chronic lymphocytic leukemia (CLL), mantle cell lymphoma (MCL) and Waldenstrӧm’s macroglobulinemia (WM) due to its efficacy. Despite the significant favourable impact in the hematologic conditions, Ibrutinib increases significantly the risk of atrial fibrillation (AF) [1]. The mechanisms leading to AF are not well established, but it is known that arterial hypertension is associated with increased risk of this dysrhythmia [2]. Yet, the safety data from ibrutinib ‘s trials regarding reported adverse events of hypertension is heterogenous.

Therefore, we performed a systematic review of randomized controlled trials to evaluate the impact of ibrutinib in the incidence of reported hypertension and atrial fibrillation on randomized controlled trials, irrespective of the comparators (active or placebo) or population.

## Methods

In this study, we performed systematic review with meta-analysis which is a well-known method to evaluate specific safety aspects of drugs using the cumulative evidence from clinical trials [3, 4].

### Search methods

We made an electronic database search through MEDLINE, EMBASE, Central Register of Controlled Trials (CENTRAL) and ClinicalTrials.gov in August 2018 and updated in December 2018 using standardized methods [5, 6] We also performed extensive hand searching by screening references of included studies and review articles for additional citations.

### Studies selection criteria

We considered eligible all randomized controlled trials (RCTs) comparing ibrutinib with any control group (placebo, no-treatment or standard care, non-pharmacological interventions or any active drug). All RCTs were considered for inclusion irrespective of patients’ baseline conditions, background therapy, ibrutinib dose, study follow-up or language of publication.

The primary outcomes were the incidence of hypertension and atrial fibrillation. For both outcomes we used a broad and lenient definition of the conditions. Hypertension was defined as blood pressure increase reported by investigators as an adverse event (or serious adverse event). AF was defined as a rhythm disorder characterized by the presence of irregular RR intervals and no discernible, distinct P waves during at least 30 seconds by convention [7], or AF reported by investigators as an adverse event. Whenever possible the adverse events were reported according to the Common Terminology Criteria for Adverse Events (CTCAE) [8].

We considered reasonable our lenient inclusion criteria as ibrutinib was only studied in a few hematologic conditions (CLL, MCL, WM) and it is not expected that the risk for hypertension or atrial fibrillation is different amongst diseases. Furthermore, our inclusive criteria increase the power of findings.

### Data extraction, evaluation, synthesis and analysis

The records retrieved through electronic database search were screened independently by 2 authors. Suitable studies were evaluated for the inclusion in the review through full-text assessment. Study selection and data extraction were performed independently. If different data were available for the same trial, we considered the most recent report or the updated data from ClinicalTrials.gov.

Study characteristics and results were extracted independently into a standardized form. Whenever possible modified intention-to-treat (patients randomized and treated at least once with the allocated drug) data was extracted for analysis.

Risk of bias was evaluated through the Cochrane Risk of Bias Tool. Disagreements throughout this process were resolved by consensus.

The incidence of hypertension or atrial fibrillation was treated as a dichotomous data and Risk Ratio (RR) was estimated. The 95% confidence interval (95%CI) was used to estimate the precision of pooled results from studies.

The data analysis was performed through the Hartung-Knapp-Sidik-Jonkman (HKSJ) method in using R 3.5.2, OpenMetaAnalyst and OpenMEE [9–11]. A secondary analysis was performed using the Mantel-Haenszel method and random effects models through RevMan version 5.3 (The Nordic Cochrane Centre, Copenhagen; The Cochrane Collaboration, 2014). A fixed 0.5 correction was added when one arm presented zero-events to avoid computational problems. Heterogeneity was assessed using the Chi square test and I^2^ statistic. The I^2^ statistics measures the percentage of total variation between studies attributed to interstudy heterogeneity rather than random [12], and we used the Sidik-Jonkman estimator to derive tau^2^ and subsequently I^2^. Statistical heterogeneity was considered substantial if I^2^> 50%.

The 95% prediction intervals were estimated to assess dispersion of the effect size in different setting, deriving whether true effects are to be expected for 95% of similar newly conducted study [11, 13, 14]. The 95% prediction intervals were put into perspective with the results of pooled analyses, using blue rectangles in a plot generated by RevMan.

We performed a subgroup analysis according to the type of control group (active or placebo). The impact of follow-up time / drug exposure in the risks of hypertension or atrial fibrillation was also ascertained through Hartung-Knapp method for meta-regression using follow-up time (months) as a covariate.

Reporting/Publication bias tests for funnel plot asymmetry were only used if a minimum of 10 studies were included in the meta-analysis [15, 16].

### Assessment of confidence in cumulative evidence

As recommended by the Grading of Recommendations Assessment, Development and Evaluation (GRADE) Working Group methodology, two reviewers independently assessed all the critical outcomes in the following domains: risk of bias, inconsistency, indirectness, imprecision and publication bias [17, 18].

## Results

**Fig 1:**
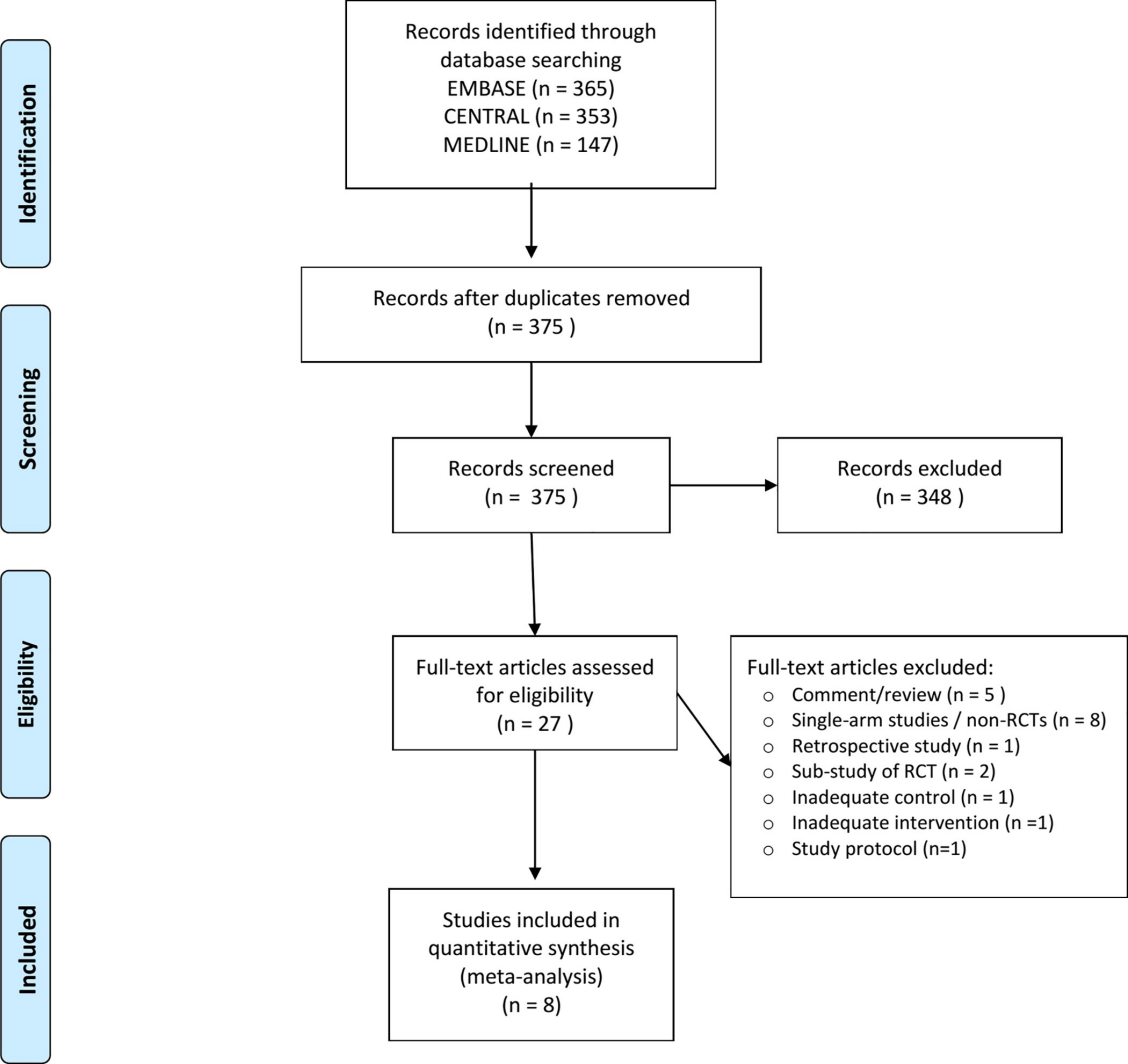
Flowchart of studies selection.

**Fig 2:**
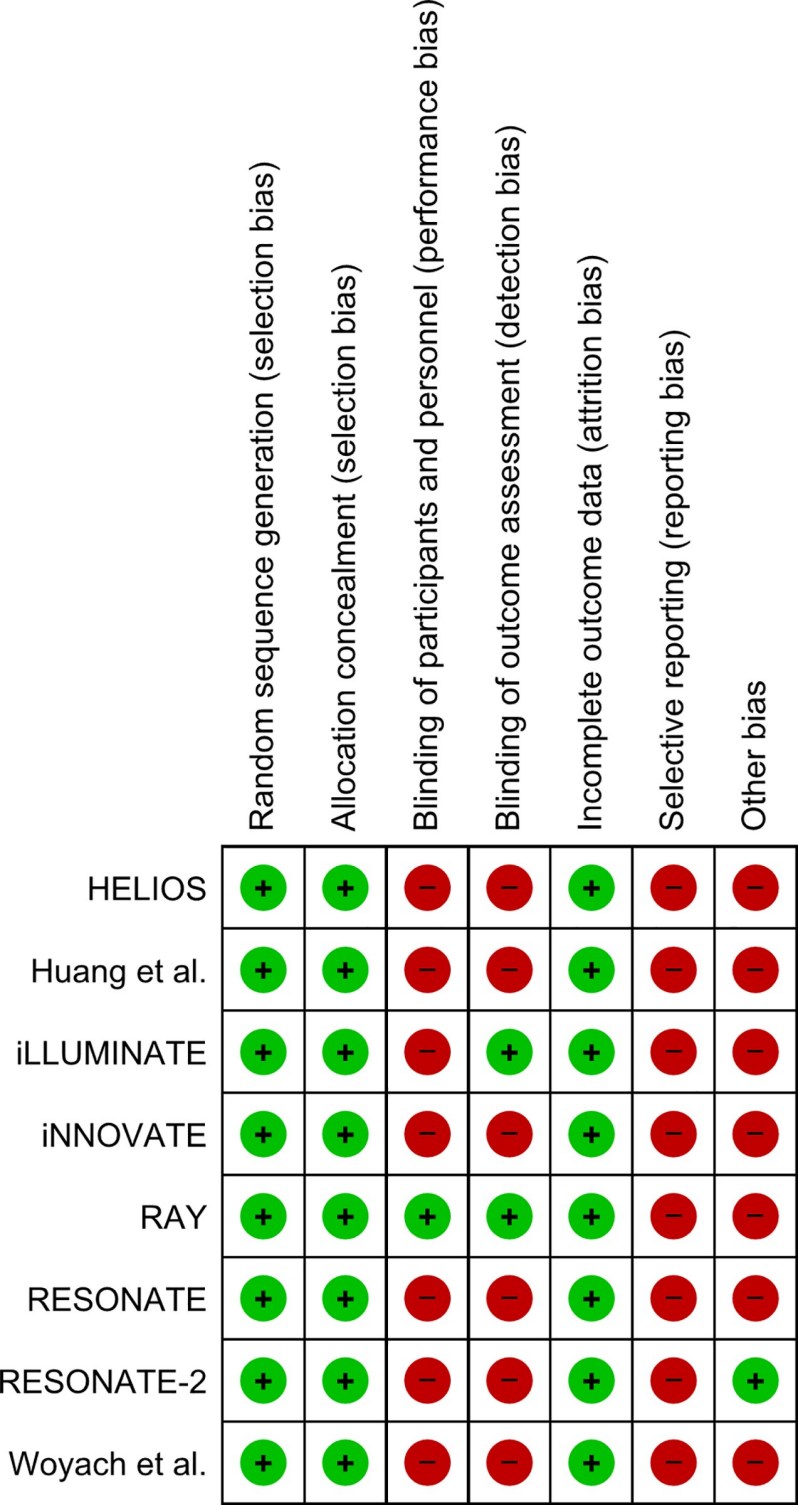
Risk of bias plot of included studies.

We found 8 eligible RCTs with 2580 patients (54.7% treated with ibrutinib) (Fig 1) [19–26]. The trial with the smaller sample size had 150 patients (iNNOVATE) and the largest had 578 patients (HELIOS). All the trials, with exception of the RAY trial [22, 27] had an open-label design (Fig 2). There were 2 placebo-controlled trials and 6 active controlled studies. None of the trials was designed for systematically search for hypertension or atrial fibrillation (high risk of selective reporting bias) (Fig 2). All the trials allowed crossovers from control to ibrutinib arm, with rates reaching 40% as occurred in iNNOVATE study [24]. Only the RESONATE-2 trial did not allow crossovers during the study (low risk of bias in ‘Other bias’ in Fig 2). The main characteristics of the included studies are depicted in Table 1 and the risk of bias plot in Fig 2 and Figure A in S1 File. Blood pressure measurements frequency was reported in half of the studies, only 3 studies reported the when ECG were performed (Table 1). About 75% of the studies reported the adverse events using CTCAE (Table 1).

**Table 1:** Included RCTs and major characteristics of the studies and patient population.

Reference	Disease	Study arms	Ibrutinib dose	No. of patients (Ibrutinib vs Placebo arm)	Patient population	Median age, y	Median Follow up, mo	Median PFS, mo	ORR, % (% CR)	Crossover allowed	BP measurement	ECG	Reporting using CTCAE
**Chanan-Khan**[21] ***HELIOS***	CLL	R-Bendamustine + Ibrutinib vs R-Bendamustine + Placebo	420mg	578 (289 vs 289)(289 in each arm)	R/R CLL ≥ 2 lines	64 vs 63	17	NR vs 13.3	82.7 (10.4) vs 67.8 (28)	Yes	N/R	N/R	N/R
**Dreyling**[22, 27] ***RAY***	MCL	Ibrutinib vs Temsirolimus	560mg	280 (139 vs 141)	R/R MCL ≥ 1 line	68	20/36	14.9 vs 6.2	72 (19) vs 40 (1)	Yes	N/R	N/R	Yes
**Dimopoulos**[24] ***iNNOVATE***	WM	Ibrutinib + Rituximab vs Placebo + Rituximab	420mg	150 (75 in each arm)	1st line and R/R	69	26.5	NR vs 20.3	92 vs 47; VGPR: 23 vs 4	Yes	Screening; Every 4 weeks until W16; every 8 weeks until end of treatment	Screening; Then ECGs should be performed at the investigator’s discretion.	Yes
**Byrd**[19] ***RESONATE***	CLL	Ibrutinib vs Ofatumumab	420mg	391 (195 vs 196)	R/R ≥ 1 line and not candidates for purine analog (including del17p)	67	9.4	NR vs 8.1	63 vs 4	Yes	Screening W1, W4, W8, every 4 weeks until W24, 12–12 weeks	Screening	Yes
**Burger**[20] ***RESONATE-2***	CLL	Ibrutinib vs Chlorambucil	420mg	269 (136 vs 133)	1st line	73	18.4	NR vs 18.9	86 (4) vs 35 (2)	No*	Screening; Day 1 and day 15 in cycles 1–7, Day 1 in cycles 8–12; Then Day 1 of cycles 13, 15, 17, 19, 21, 23, 25), and every 6 cycles beginning in Cycle 30	Screening; Then ECGs should be performed at the investigator’s discretion.	Yes
**Huang**[23]	CLL	Ibrutinib vs Rituximab	420mg	160 (106 vs 54)	R/R ≥ 1 line and not candidates for purine analog	66	17.8	NR vs 8.3	53.8 (3.8) vs 7.4 (0)	Yes	N/R	N/R	Yes
**Woyach**[25]	CLL	Ibrutinib vs Ibrutinib + Rituximab vs Bendamustine + Rituximab	420 mg mg	576 (180 vs 181 vs 176)	1st line	71	43	NR vs 43	93 (7) vs 94 (12) vs 81 (26)	Yes	Screening; Day 1 of Cycles 2–6; Day 1 of Every Third Cycle during treatmentCycle During Treatment	N/R	Yes
**Moreno**[26] **iLLUMINATE**	CLL	Ibrutinib + obinutuzumab vs chlorambucil + obinutuzumab	420 mg	229 (113 vs 116)	1st line	71	31	NR vs 19	88 (19) vs 73 (8)	Yes	N/R	N/R	Yes

PFS indicates Progression Free Survival assessed by independent review committees; ORR, Overall Response Rate; CR, Complete Response; CLL, Chronic Lymphocytic Leukemia; CTCAE, Common Terminology Criteria for Adverse Events; R/R, Relapsed/refractory disease; NR, Not Reached; N/R, not reported; MCL, Mantle-cell Lymphoma; WM, Waldenstrom’s Macroglobulinemia; VGPR, Very Good Partial Response

*Patients can receive Ibrutinib as part of a separate extension study, according to the investigator’s choice

### Risk of hypertension with ibrutinib

The pooled analysis included 8 studies. Half of the studies, individually, did not reach the statistical significance.

**Fig 3:**
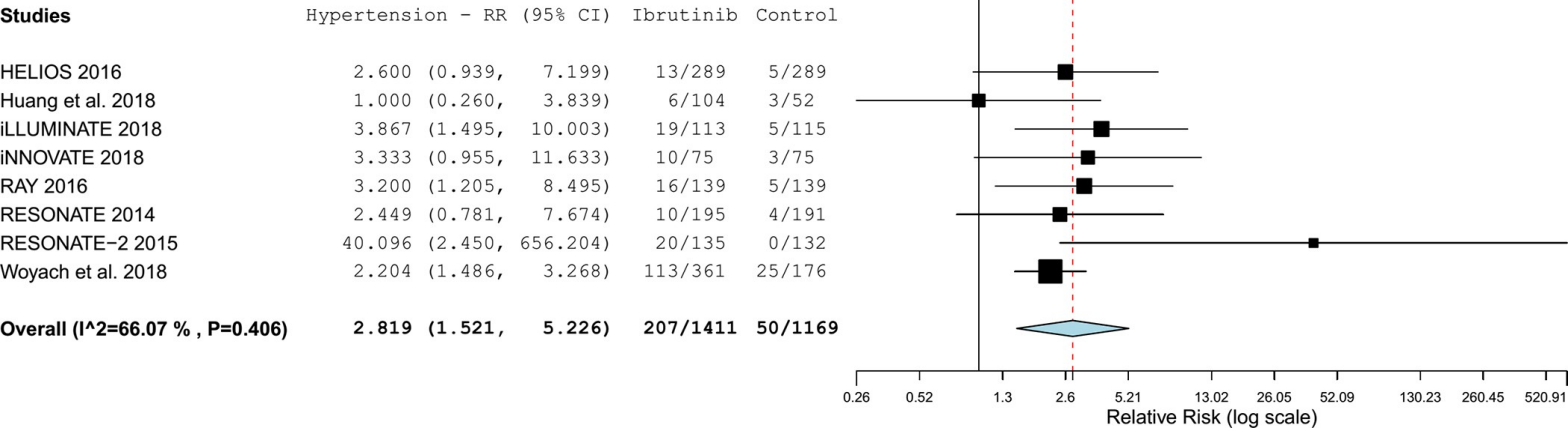
Forest plot with risks of hypertension associated with ibrutinib.

**Fig 4:**
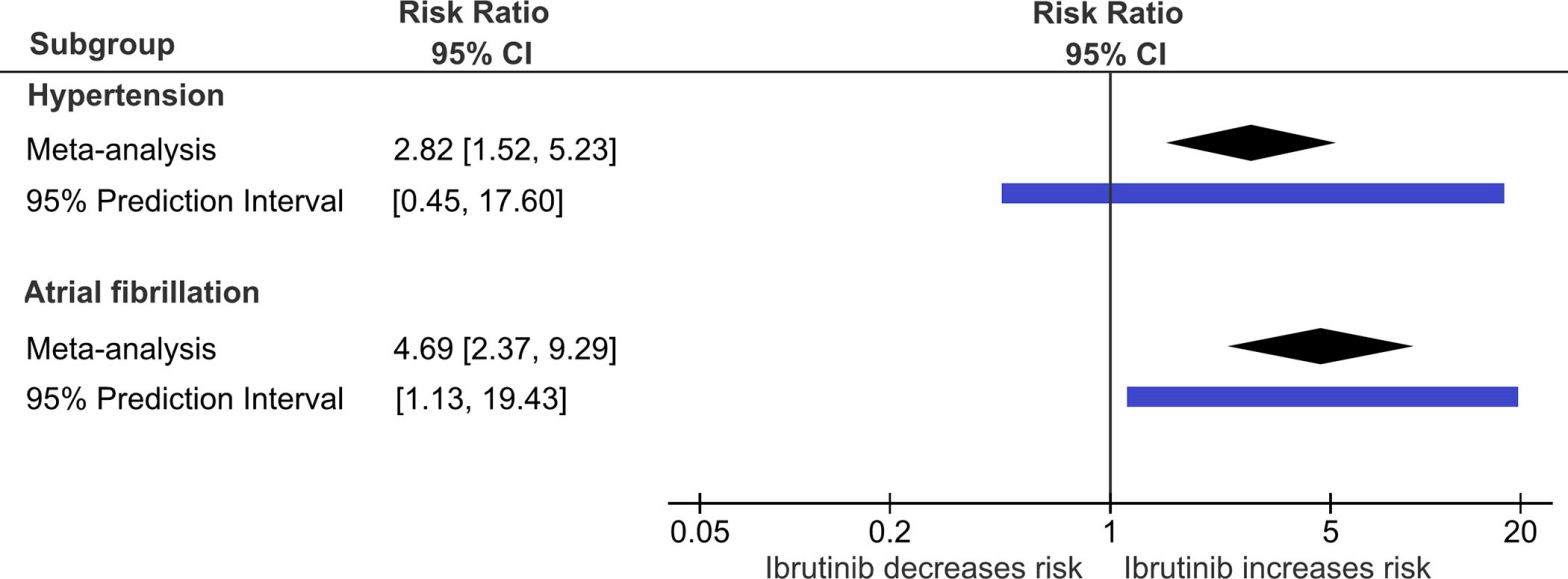
Risks of hypertension and atrial fibrillation associated with ibrutinib from meta-analysis (black diamond) and 95% predictive intervals (blue rectangle).

Overall the pooled estimate showed that ibrutinib increased significantly the risk of hypertension with a RR of 2.82 (95%CI 1.52–5.23; p-value <0.001) (Fig 3 and Fig 4) and substantial statistical heterogeneity was observed (tau^2^ = 0.461; I^2^ = 66%). The 95% prediction interval ranged from 0.45 to 17.60 (Fig 4).

The small number of studies found (<10) preclude the use of reporting/publication bias tests and funnel plot interpretation (Figure B in S1 File).

### Risk of atrial fibrillation with ibrutinib

**Fig 5:**
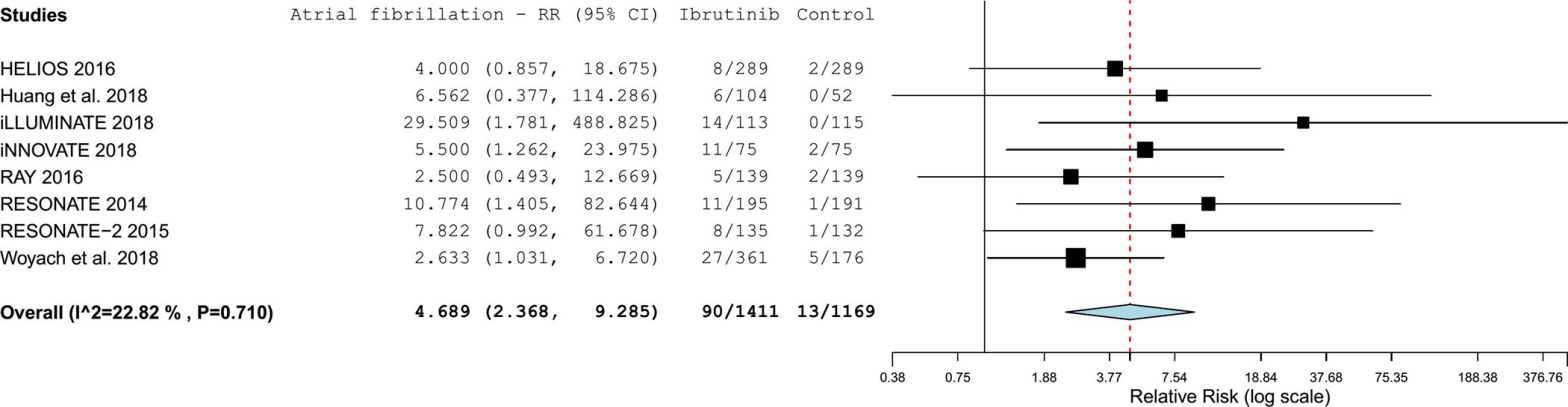
Forest plot with risks of atrial fibrillation associated with ibrutinib.

The pooled analysis included 8 studies. Four of them showing significant increases in the risk of atrial fibrillation. The pooled risk ratio showed more than 4-fold increase in the risk of AF with ibrutinib (RR = 4.69, 95%CI 2.17–7.64; p-value <0.001) (Fig 4 and Fig 5). The 95% prediction interval ranged from 1.13 to 19.43 (Fig 4).

The analysis was unremarkable for substantial statistical heterogeneity (tau^2^ = 0.216; I^2^ = 22.8%). The Figure C in S1 File shows the funnel plot for this outcome, but the reporting/publication bias tests and plot interpretation were not formally performed due to small number of studies (<10).

### Additional analyses

The subgroup analyses according with the control group (active control / placebo) did not show any significant interactions with risk estimates (Figure D in S1 File).

The meta-regression using follow-time as a covariate did not show a significant relationship between hypertension risk (p = 0.93) nor atrial fibrillation risk (p = 0.158) with ibrutinib exposure time (Figure E and Figure F in S1 File). The meta-analyses using the Mantel-Haenszel method and random effects models is shown in Figure G in S1 File, and the results are globally similar to the primary analyses.

### Grading of recommendations assessment, development and evaluation (GRADE)

We used the GRADE approach to ascertain the confidence of the obtained pooled results. Both hypertension and atrial fibrillation were analysed through the same trials. Overall, we considered that the results may have been influenced by performance bias and selective reporting bias (Fig 2). Even though the results were consistent, and the estimates were large for hypertension and very large for atrial fibrillation. This determined a moderate and high certainty in the pooled evidence for hypertension and atrial fibrillation risk with ibrutinib, respectively. The GRADE evaluation with absolute and relative risk increases are detailed in Table 2.

**Table 2:** Summary of findings table–GRADE Approach.

Risk of hypertension and atrial fibrillation associated with Ibrutinib						
Intervention: Ibrutinib; Comparison: Control						
Outcomes	Anticipated absolute effects* (95% CI)		Relative effect (95% CI)	№ of participants (studies)	Certainty of the evidence (GRADE)	Comments
	Risk with Control	Risk with Ibrutinib				
Hypertension	4 per 100	**12 per 100** (7 to 22)	**RR 2.82** (1.52 to 5.23)	2580 (8 RCTs)	⨁⨁⨁○ MODERATE	All studies had high-risk of bias due to selective reporting and only one trial had adequate blinding. Ibrutinib probably results in a large increase in hypertension risk.
Atrial fibrillation	1 per 100	**5 per 100** (2 to 8)	**RR 4.69** (2.17 to 7.64)	2580 (8 RCTs)	⨁⨁⨁⨁ HIGH	All studies had high-risk of bias due to selective reporting and only one trial had adequate blinding. The very large effect documented consistent (5 RCTs with RR>5.0) upgraded the quality of evidence, despite the risk of bias Ibrutinib is likely to results in a very large increase in atrial fibrillation risk.

*The risk in the intervention group (and its 95% confidence interval) is based on the assumed risk in the comparison group and the **relative effect** of the intervention (and its 95% CI). **CI:** Confidence interval; **RR:** Risk ratio

**GRADE Working Group grades of evidence**

**High certainty:** We are very confident that the true effect lies close to that of the estimate of the effect

**Moderate certainty:** We are moderately confident in the effect estimate: The true effect is likely to be close to the estimate of the effect, but there is a possibility that it is substantially different

**Low certainty:** Our confidence in the effect estimate is limited: The true effect may be substantially different from the estimate of the effect

**Very low certainty:** We have very little confidence in the effect estimate: The true effect is likely to be substantially different from the estimate of effect.

## Discussion

In this systematic review with meta-analysis, we showed that ibrutinib was significantly associated with an increased risk of hypertension in randomized controlled trials. Hypertension was considered a common adverse event of ibrutinib in the Summary of Product Characteristics of ibrutinib [28], but the data was solely derived from the rates of reporting in clinical studies or during post marketing surveillance without a formal comparison with a control arm. In this systematic review we also confirmed with new published trials that ibrutinib was associated with atrial fibrillation.

The major contributions for the current knowledge are: 1) the confirmation of hypertension as adverse drug reaction of ibrutinib; 2) the updated meta-analysis showing the association between ibrutinib and atrial fibrillation with more trials (8 RCTs) and more precision and confidence on the estimate compared with other published meta-analyses [1, 29, 30]; 3) The use of prediction intervals to determine that atrial fibrillation risk is likely to be increased but hypertension risk may not be increased in new studies, taking into account the dispersion of data; 4) the use of GRADE approach to include all aspects of the trials and analyses to ascertain the confidence on the new data supports that the confidence of AF increased risk is high and moderate for increase of hypertension risk.

As a secondary effect of ibrutinib, hypertension needs to be treated to mitigate cardiovascular consequences, such as atrial fibrillation, myocardial infarction or stroke.

One of the reasons for this study was the known link between hypertension and atrial fibrillation. Despite the heterogeneous results of studies evaluating the association of hypertension and AF in patients treated with ibrutinib, the link of hypertension and atrial fibrillation is well established [1, 31]. Being hypertension a risk factor for AF, an adequate diagnosis and treatment of this risk factor may be beneficial as hypertension and AF are linked to stroke [32–34]. The risk ratios of hypertension and atrial fibrillation associated with ibrutinib were different (RR 2.82 hypertension, RR 4.69 for atrial fibrillation) and preclude an absence of direct association. The absolute risk (Table 2) shows that ibrutinib increases risk of hypertension in 7.8% (12 new cases for 100 patients treated) and atrial fibrillation in 4.1% (5 new cases for 100 patients treated). This paradoxus (lower RR and higher absolute risk for hypertension compared with AF) is explained by the higher prevalence and reported adverse event of hypertension compared with atrial fibrillation.

Beyond the putative role of hypertension-linked AF, the inhibition of the PI3K-Akt pathway by ibrutinib may also play a pathogenic role in the development of AF, but it is still controversial [35–37].

One of the most important limitations is that none of the included studies was designed to detect / adjudicate incident or worsening of hypertension and atrial fibrillation. The confidence on the current evidence is at least moderate, and the future trials with ibrutinib will surely acknowledge the blood pressure and atrial fibrillation issues. At outcome level, the subjective nature of hypertension reporting by investigators should be noticed as at high-risk of selective reporting bias. The diagnosis of AF is more objective, but it also depends on clinical manifestations, and it is known that AF can be clinically silent (until the embolic consequences) and/or paroxysmal, which may impair an accurate reporting.

The concomitant increase in the risks of AF and hypertension, together with the increased bleeding risk of ibrutinib is a clinical challenge. In patients with AF and one thromboembolic risk factor (such as hypertension), the stroke risk is considered non-negligible and the guidelines say that anticoagulation should be considered [7, 38, 39]. Thus, AF with ibrutinib, thromboembolic risk factors (hypertension) and bleeding risk factors (antiplatelet effect of ibrutinib and anticoagulation) may pose difficulties to the clinicians.

This systematic review was not able to capture differences the clinical consequences and severity of hypertension and atrial fibrillation, that vary widely. An unanswered relevant question for the clinical practice is the degree of blood pressure increase induced by ibrutinib and its relevance for the development of other consequences.

Our results are important for clinicians and investigators as they emphasize that other cardiovascular events / risk factors associated with ibrutinib, such as arterial hypertension, should be targeted in further investigations.

The efficacy of ibrutinib poses new questions as these data are derived from the trials reveal an increase of patients’ survival. Studies with longer follow-up are required to better characterize the risk at longer term, as well as other clinical aspects such as the carry-over effect, the durability of the adverse events, and the management of hypertension and/or atrial fibrillation with different strategies [40–44].

### Limitations

Results and conclusion here presented are weakened by limitations inherent to meta-analysis and individual studies [45, 46].

The higher risk of bias was found for potential selective reporting of the outcomes, i.e. the outcomes were not primary and actively searched, and were reported at the discretion of the investigator [47]. The results might have been also influenced by the risk of performance bias (lack of blinding) which limits the association of the adverse events with ibrutinib. A limitation is that there is not a single RCT primarily designed to evaluate hypertension and atrial fibrillation as primary outcomes. Nonetheless, the degree of confidence on the current evidence is moderate/high.

The non-negligible rates of crossovers from control arms to ibrutinib (when disease progression occurred in control arm) may limit our analysis. However, the analysis of the data as intention-to-treat increases the confidence in our results as some control patients could have developed hypertension and/or atrial fibrillation after crossing to the ibrutinib arm and were analysed as control. Thus, despite this conservative approach, our results were significant.

## Conclusions

Our results suggest that ibrutinib increases significantly the risks of hypertension and atrial fibrillation. The pooled data for hypertension had moderate quality evidence, and high-quality evidence for atrial fibrillation. Whether hypertension is a crucial factor for ibrutinib-induced atrial fibrillation is still unknown.

## Supporting information

S1 File Supplementary data. (PDF)

S2 File PRISMA checklist. (PDF)
