# Supplementary material for: Ibrutinib increases the risk of hypertension and atrial fibrillation: Systematic review and meta-analysis
Source: PLoS One. 2019 Feb 20;14(2):e0211228. doi: 10.1371/journal.pone.0211228 (PMC6382095; doi:10.1371/journal.pone.0211228)
Supplement: S1 File — (PDF) [file pone.0211228.s001.pdf]

SUPPLEMENTARY DATA

PLOS ONE

Ibrutinib increases the risk of hypertension and atrial fibrillation: systematic review and meta-analysis.

.

Daniel Caldeira, Daniela Alves, João Costa, Joaquim J. Ferreira, Fausto J. Pinto.

Corresponding author: Prof. Daniel Caldeira, Faculdade de Medicina da Universidade de Lisboa, Av. Prof. Egas Moniz, Lisboa, 1649-028, Portugal. E-mail: dgcaldeira@hotmail.com

Phone number: (+351) 21 797 34 53; **Fax number:** (+351) 21 781 96 88.

Contents

Search Strategies 3

Figure A: Overview of trials’ risk of bias. 4

Figure B: Funnel plot for publication bias evaluation – hypertension. 5

Figure C: Funnel plot for publication bias evaluation – atrial fibrillation. 6

Figure D: Forest plot of risk associated with ibrutinib according to the controls used in the trials. 7

Figure E: Meta-regression evaluating the association of hypertension risk and follow-up 8

Figure F: Meta-regression evaluating the association of atrial fibrillation risk and follow-up 9

Figure G: Forest plot with meta-analyses using the Mantel-Haenszel method and random effects models. 10

Search Strategies

Ovid MEDLINE

#	Searches
1	ibrutinib.af.
2	randomized controlled trial.pt.
3	controlled clinical trial.pt.
4	randomized.ab.
5	placebo.ab.
6	randomly.ab.
7	clinical trials as topic.sh.
8	trial.ti.
9	2 or 3 or 4 or 5 or 6 or 7 or 8
10	exp animals/ not humans.sh.
11	9 not 10
12	1 and 11

EMBASE

#	Searches
1	ibrutinib.
2	random*:ab,ti OR placebo*:de,ab,ti OR (double NEXT/1 blind*):ab,ti.
3	1 and 2

Ovid CENTRAL

#	Searches
1	ibrutinib.af.

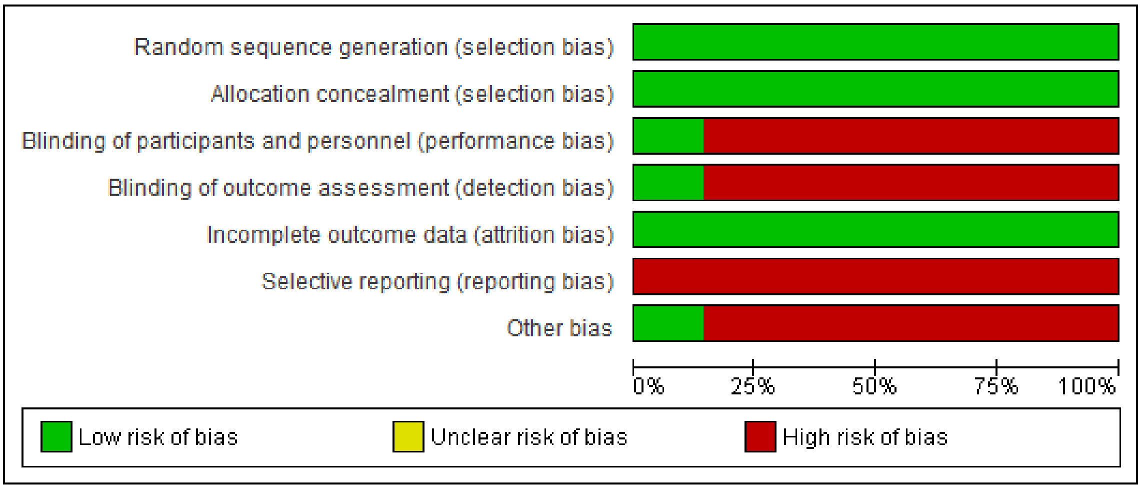

Figure A: Overview of trials' risk of bias.

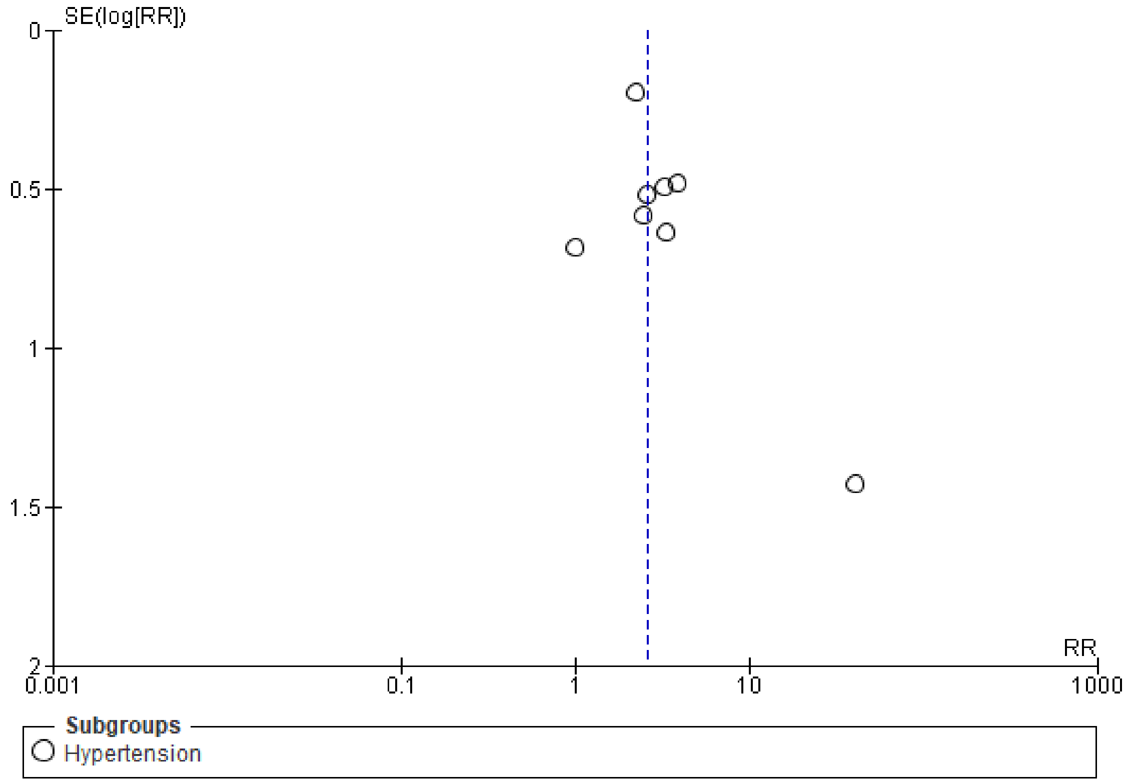

Figure B: Funnel plot for publication bias evaluation – hypertension.

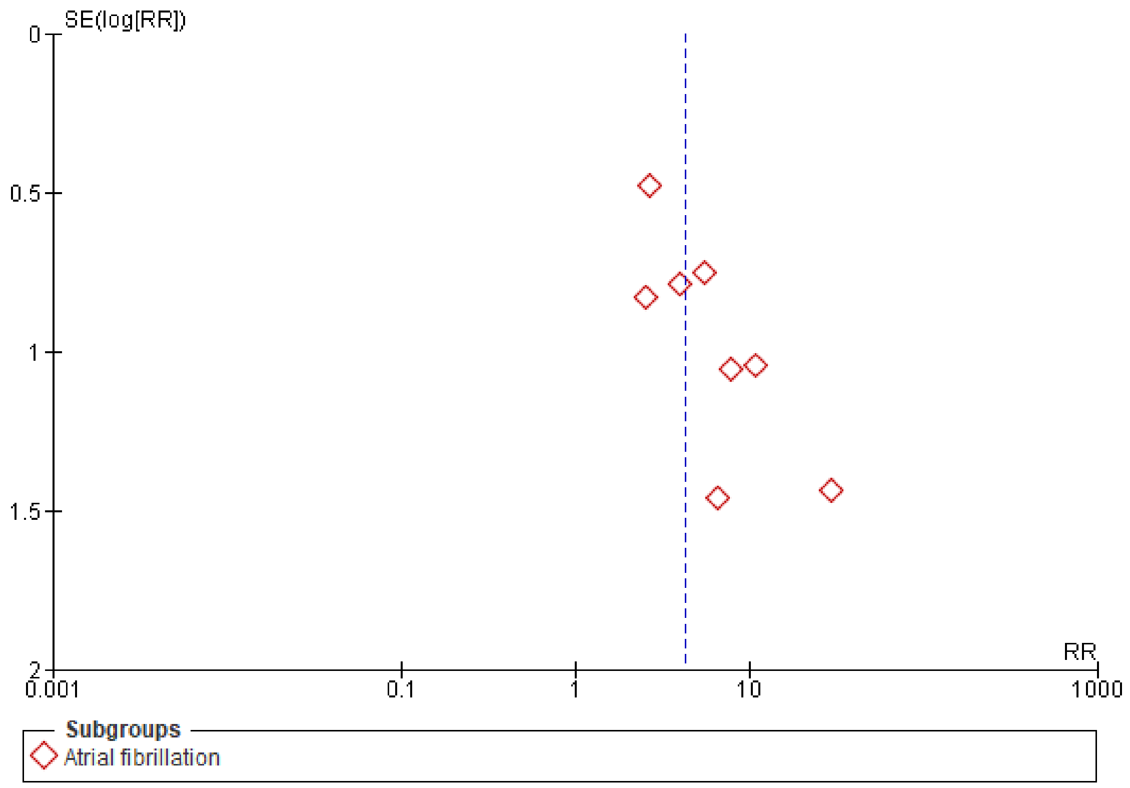

Figure C: Funnel plot for publication bias evaluation – atrial fibrillation.

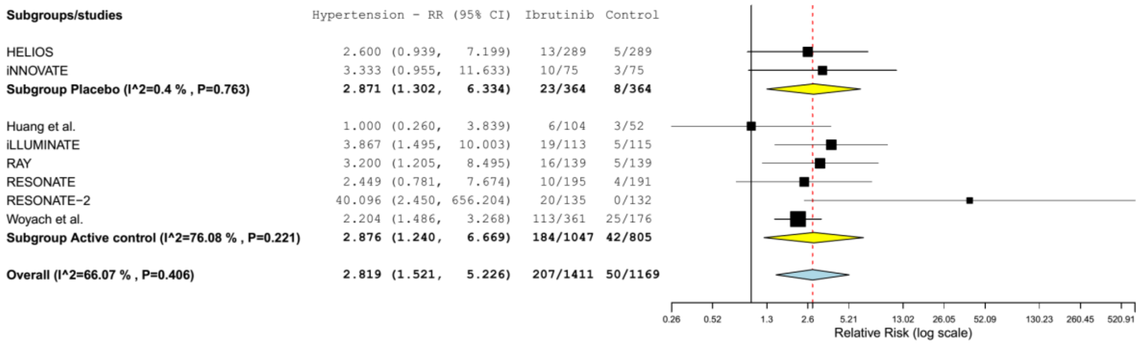

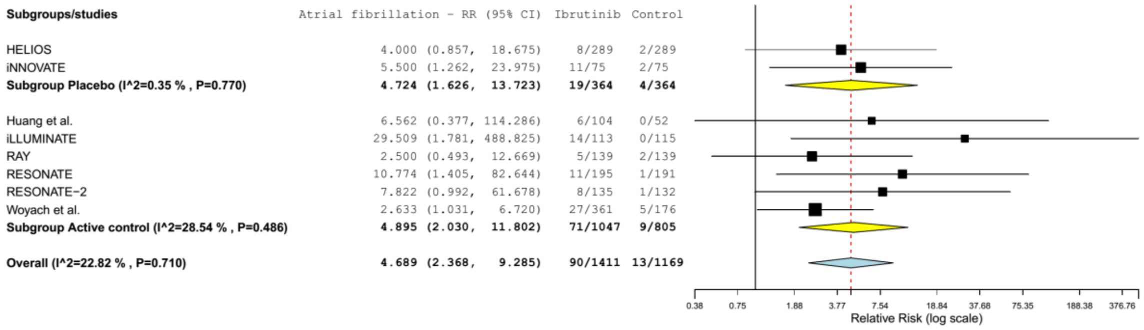

Figure D: Forest plot of risk associated with ibrutinib according to the controls used in the trials. P-value for interaction between groups regarding hypertension: p=0.99; P-value for interaction between groups regarding atrial fibrillation: p=0.96.

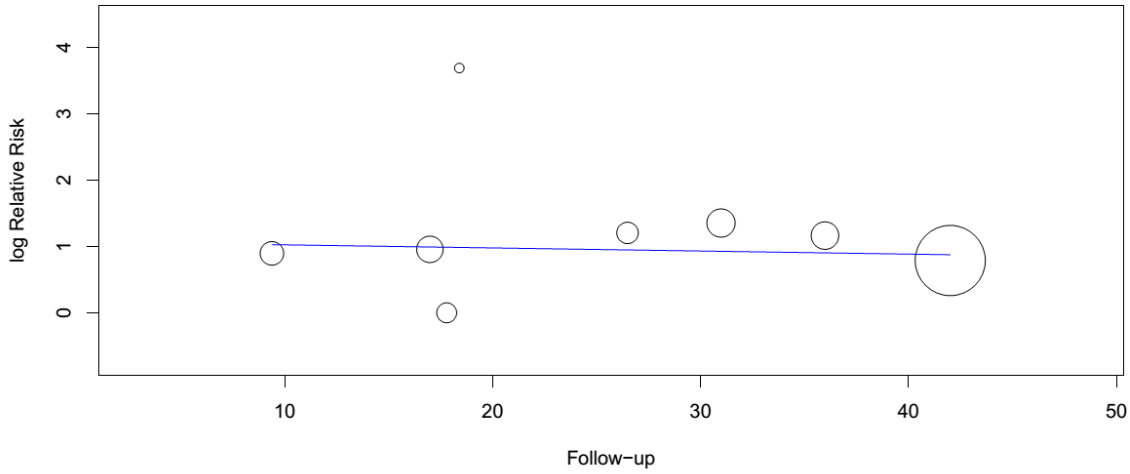

Figure E: Meta-regression evaluating the association of hypertension risk and follow-up (p=0.93).

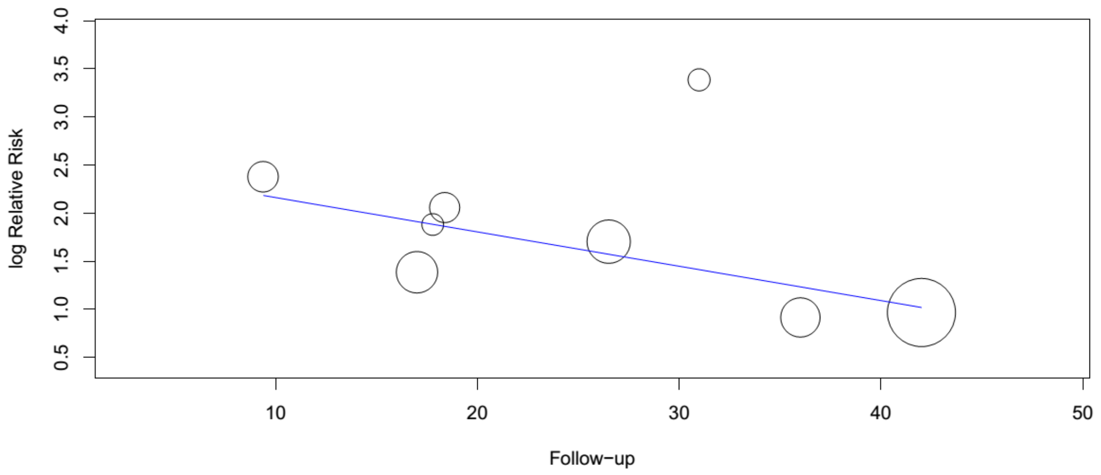

Figure F: Meta-regression evaluating the association of atrial fibrillation risk and follow-up (p=0.158).

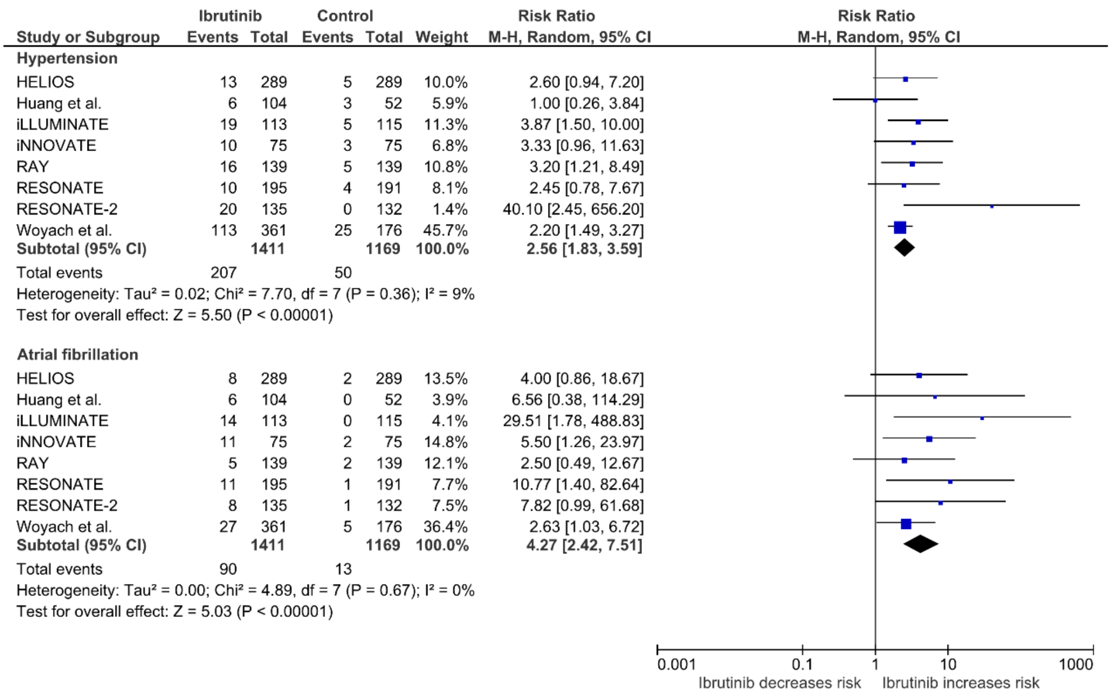

Study or Subgroup	Ibrutinib Events	Ibrutinib Total	Control Events	Control Total	Weight	Risk Ratio M-H, Random, 95% CI
**Hypertension**						
HELIOS	13	289	5	289	10.0%	2.60 [0.94, 7.20]
Huang et al.	6	104	3	52	5.9%	1.00 [0.26, 3.84]
iLLUMINATE	19	113	5	115	11.3%	3.87 [1.50, 10.00]
iNNOVATE	10	75	3	75	6.8%	3.33 [0.96, 11.63]
RAY	16	139	5	139	10.8%	3.20 [1.21, 8.49]
RESONATE	10	195	4	191	8.1%	2.45 [0.78, 7.67]
RESONATE-2	20	135	0	132	1.4%	40.10 [2.45, 656.20]
Woyach et al.	113	361	25	176	45.7%	2.20 [1.49, 3.27]
**Subtotal (95% CI)**		**1411**		**1169**	**100.0%**	**2.56 [1.83, 3.59]**
Total events	207		50			
Heterogeneity: Tau² = 0.02; Chi² = 7.70, df = 7 (P = 0.36); I² = 9%						
Test for overall effect: Z = 5.50 (P < 0.00001)						
**Atrial fibrillation**						
HELIOS	8	289	2	289	13.5%	4.00 [0.86, 18.67]
Huang et al.	6	104	0	52	3.9%	6.56 [0.38, 114.29]
iLLUMINATE	14	113	0	115	4.1%	29.51 [1.78, 488.83]
iNNOVATE	11	75	2	75	14.8%	5.50 [1.26, 23.97]
RAY	5	139	2	139	12.1%	2.50 [0.49, 12.67]
RESONATE	11	195	1	191	7.7%	10.77 [1.40, 82.64]
RESONATE-2	8	135	1	132	7.5%	7.82 [0.99, 61.68]
Woyach et al.	27	361	5	176	36.4%	2.63 [1.03, 6.72]
**Subtotal (95% CI)**		**1411**		**1169**	**100.0%**	**4.27 [2.42, 7.51]**
Total events	90		13			
Heterogeneity: Tau² = 0.00; Chi² = 4.89, df = 7 (P = 0.67); I² = 0%						
Test for overall effect: Z = 5.03 (P < 0.00001)						

Figure G: Forest plot with meta-analyses using the Mantel-Haenszel method and random effects models.
